# First Record of *Triatoma maculata* (Erichson, 1848) (Hemiptera: Reduviidae: Triatomini) in the Municipality of Riohacha, La Guajira – Colombia

**DOI:** 10.3389/fpubh.2014.00219

**Published:** 2014-11-10

**Authors:** Edith Natalia Gómez-Melendro, Carolina Hernández, Catalina González-Uribe, Helena Brochero

**Affiliations:** ^1^Programa ECOSALUD ETV Colombia, Centro de Estudios e Investigación en Salud (CEIS), Fundación Santa Fe de Bogotá, Bogotá, Colombia; ^2^Red Chagas Colombia, Grupo Parasitología, Instituto Nacional de Salud, Bogotá, Colombia; ^3^Facultad de Ciencias Agrarias, Universidad Nacional de Colombia, Bogotá, Colombia

**Keywords:** Chagas disease, vector indices, Wayúu ethnicity, Triatomini

## Abstract

**Introduction:** Knowledge of vector insect species, their habitat, and geographical distribution is crucial for determining the risk of transmission of the etiological agents that cause disease in humans, which allows defining strategies for prevention, surveillance, and control in line with the characteristics of each area.

**Objective:** To determine the presence and public health importance of vectors of Chagas disease in the indigenous settlements of Marbacella and El Horno of the Wayúu ethnic group in the municipality of Riohacha, La Guajira, Colombia.

**Materials and Methods:** From active search, installation and inspection of biosensors, and occasional catches, Hemiptera: Reduviidae: Triatomini were collected intra and in the peridomicile housing of the indigenous settlements of El Horno and Marbacella of the Wayúu ethnic group. Indices of intra and peridomestic infestation, colonization, density, dispersion, and natural infection with *Trypanosoma cruzi* Chagas, 1909 were calculated.

**Results:** 79.6% (*n* = 90) of the specimens were collected in the peridomicile and 20.3% (*n* = 23) in the intradomicile, all corresponding to *Triatoma maculata* (Erichson, 1848). The natural infection indices with *T. cruzi* accounted for 43.5% for Marbacella and 36% for El Horno.

**Conclusion:** This is the first reported capture of individuals of *T. maculata*, considered a secondary vector of Chagas disease in Colombia, naturally infected with *T. cruzi* in the municipality of Riohacha expanding the geographical distribution of the species in the department of La Guajira.

## Introduction

Chagas disease is a parasitic disease caused by *Trypanosoma cruzi* Chagas, 1909, mainly transmitted by blood-sucking insects belonging to the subfamily Triatominae (Hemiptera) (1). It is endemic in 21 countries in the Americas, and it is considered that between 18 and 20 million people are infected and 100 million are at risk of acquiring infection (2). American trypanosomiasis in Colombia is considered a public health problem because it is estimated that about eight million people are exposed to disease transmission and between 700,000 and 1,200,000 are infected with the parasite (3, 4).

In Latin America, the transmission of *T. cruzi* particularly occurs in rural areas, where triatomines have adapted to human habitat due to bioecological, political, socio-economic, and cultural factors. *Rhodnius prolixus* Stal, 1859, *Triatoma dimidiata* Latreille, 1811 and *Triatoma infestans* Klug, 1834, have represented, in terms of parasitological, epidemiological, and public health, the most important vectors in the transmission of Chagas disease (5, 6). In Colombia, *R. prolixus* and *T. dimidiata* are considered as major vectors because they are widely distributed and have high indices of house infestation, colonization, and natural infection with *T. cruzi* (4, 7, 8). However, in areas where these species are not domiciled but have had outbreaks of the disease, studies have suggested the participation of secondary vectors such as *T. maculata* (9–11), *Rhodnius pallescens* Barber, 1932 (12), *Eratyrus cuspidatus* Stal, 1859 (13) and *Panstrongylus geniculatus* Latreille, 1811 (14). *P. geniculatus* was indicted in a major acute outbreak of Chagas disease in the Colombian Orinoco in 2014 (15).

In the municipality of Riohacha, La Guajira Department were recorded *R. prolixus*, *T. maculata*, and *T. dimidiata* with house infestation indices of 12.28% and naturally infected with *Trypanosoma rangeli* Tejera, 1920 and *T. cruzi*, respectively, with the *R. prolixus* (79.27%) and *T. maculata* (23.37%) populations being the most abundant in intradomiciliary environments, suggesting that this area of the country has optimal biological and ecological factors for the presence of important epidemiological triatomine bugs in human habitats (16). Because 44.9% of the human population in this municipality is indigenous (17), mainly of the Wayúu ethnic group, there are characteristics and socio-cultural practices in rural areas, which combined with the above could increase the level of risk of vectorial transmission of *T. cruzi* in the municipality of Riohacha.

In this context, the aim of this study was to determine the presence and public health importance of vectors of Chagas disease in the indigenous settlements of Marbacella and El Horno of the Wayúu ethnic group in the municipality of Riohacha, La Guajira, Colombia.

## Materials and Methods

### Study area

**Figure 1:**
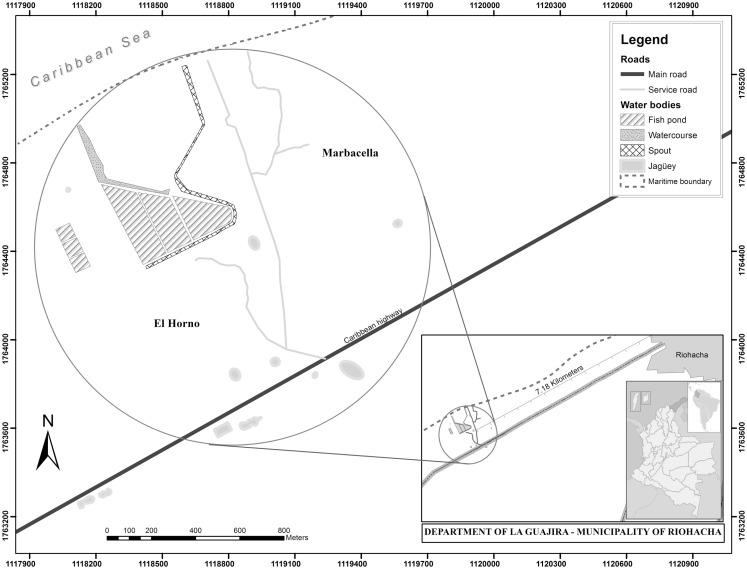
**Geographical location of El Horno and Marbacella settlements, Riohacha, La Guajira, Colombia is shown**.

The municipality of Riohacha, the capital of the department of La Guajira, is located on the Colombian Caribbean coast (Figure 1). The locations correspond to the indigenous settlements of El Horno (11°30′16.35″N and 72°59′21.31″W) and Marbacella (11°30′24.5″N and 72°59′09.7″W) situated at 7.18 km from the Riohacha city center (Figure 1). The indigenous settlements are inhabited by about 300 members of the Wayúu ethnic group who are mainly engaged in fishing, making crafts, and grazing goats. The area corresponds to Tropical Dry Forest (Bs-T) (18, 19) with preferentially herbaceous vegetation, an average annual temperature of 28.2°C (minimum 23.4°C, maximum 33.2°C), relative humidity ranging between 59.3 and 77.5%, and an annual bimodal rainfall regime, with the first rainy season between the months of April and June and another more representative period in the months of September and October, with rainfall between 700 and 2,000 mm (20–23).

### Entomological material

Three entomological surveys were performed during dry season (May, June and July 2013) and one during rainy season (September–October 2013) to search for feces, exuviae, eggs, immature, and adult forms of Hemiptera: Reduviidae: Triatomini, associated with the dwellings in the indigenous settlements of Marbacella and El Horno in Riohacha. Each sampling included direct search in each house following the National Protocols of Entomological Surveillance (4); setting of Maria sensor type traps (24); and training people in the community for triatomine recognition.

The search was performed directly inside the houses examining the furniture, appliances, clusters of objects, cracks in walls and other places with favorable conditions for the presence of these insects, and around the homes inspecting kitchens, henhouses, pens, woodpiles, shady arbors, and animal nests (4, 24). Maria sensor type traps (*n* = 20) were installed at a height of 1.50 m from the floor, on the inside walls of randomly selected houses at each sampling, participation workshops were held for residents of communities to recognize the epidemiologically relevant triatomine species reported for the area and the invitation was to actively search and deliver entomological material collected. For this, full-size photographs of insects were used as well as entomological material from colony, pin mounted, and properly labeled. Biosafety standards for handling insects were emphasized and latex gloves, masks, entomological tweezers, and labeled bottles for data collection were delivered. In all cases, the insects were collected in plastic vials labeled with the catch information for each sampling method and were transported to the Laboratory of Entomology – Genetics Area of Economic Interest Insects, Faculty of Agricultural Science, Universidad Nacional de Colombia.

The taxonomic identification of immature stages and adults was based on morphological characters, including male genital specimens (6). The determination of the natural infection with *T. cruzi* was performed by optical microscopy inspection in 0.9% saline of the content of the distal portion of the insect gut. Also parasitic DNA detection was performed by polymerase chain reaction (PCR) amplification of a satellite nuclear region with *cruzi1* primers (5′ASTCGGCTGATCGTTTTCGA3 ′) and *cruzi2* (5′AATTCCTCCAAGCAGCGGATA3′) from abdomen of representative *T. maculata* specimens of each home. PCR was performed by initial denaturation of 94°C for 5 min followed by 40 cycles of 94°C for 1 min, 64°C for 30 s, and 72°C for 1 min. The PCR products were analyzed by electrophoresis in 2% agarose gels stained with GelRed (Biotum) (25).

For each indigenous settlement the following indices were calculated: intra and peridomestic infestation; colonization; infection; dispersion; and density, all in accordance with the guidelines of the Pan American Health Organization (PAHO) (26).

### Ethical issues

The project was approved by the Research Ethics Corporate Committee of the Fundación Santa Fe de Bogotá. The insect collection was conducted by officials from the Health Office of the department of La Guajira; researchers at the Fundación Santa Fe de Bogota, and Universidad Nacional de Colombia, as well as by citizens of the community who were trained to perform these activities and signed an informed consent where they were explained the objectives, risks, and benefits of the activity. The heads of households where the direct search for triatomines and installation of Maria sensors were conducted were informed of the purpose of the study, identifying their benefits and risks and were asked to consent to the development of the activities. The results obtained were shared with the Health Office of the department and the community of the indigenous settlements of El Horno and Marbacella of the Wayúu community of Riohacha, La Guajira.

## Results

**Figure 2:**
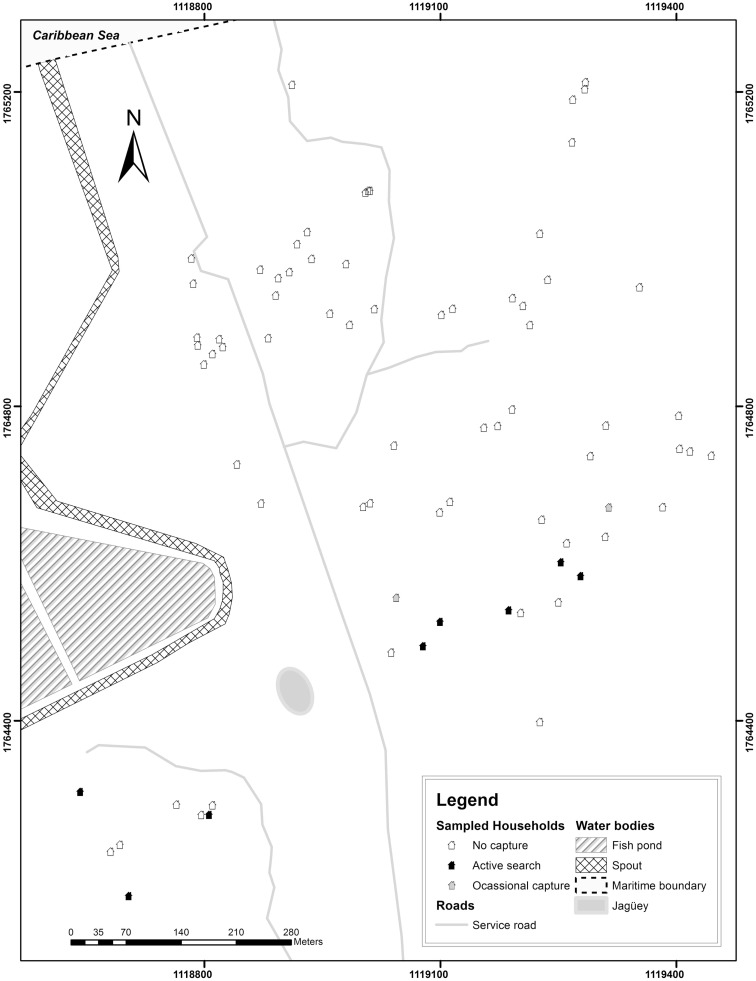
**Intradomiciliary sampling of *Triatoma maculata* (Erichson, 1848) at El Horno y Marbacella settlements, Riohacha, La Guajira, Colombia 2013 is shown**.

**Figure 3:**
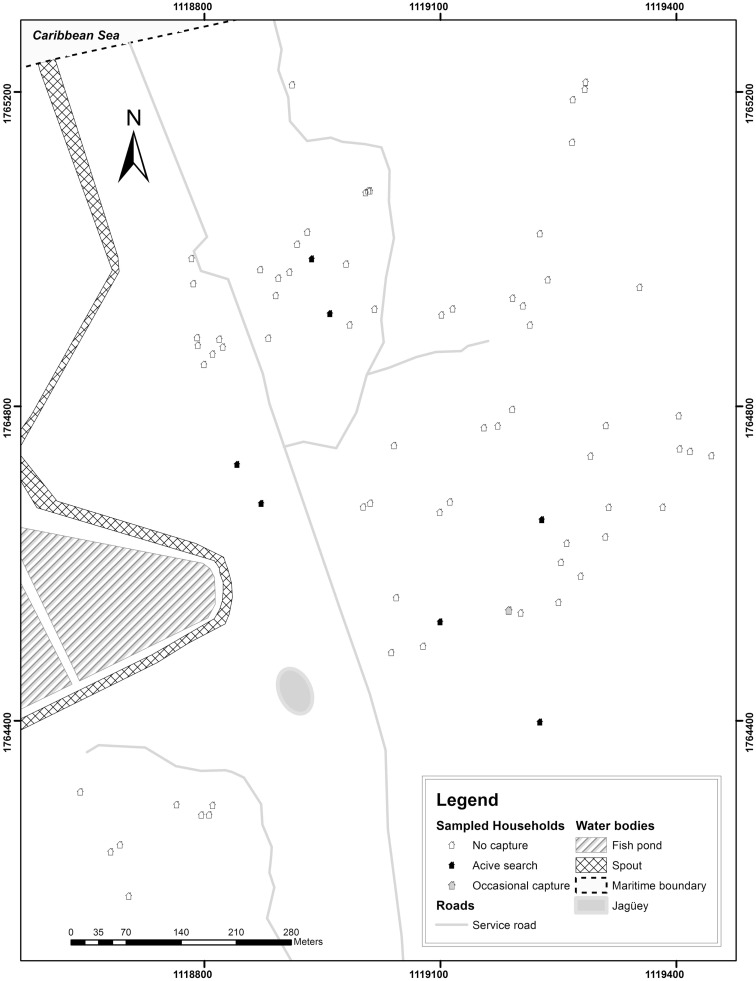
**Peridomiciliary sampling of *Triatoma maculata* (Erichson, 1848) at El Horno y Marbacella settlements, Riohacha, La Guajira, Colombia 2013 is shown**.

A total of 68 homes out of 75 (90.7%) were inspected due to the reluctance of the inspections by some heads of household. Direct search and occasional catches evidenced the presence of Hemiptera: Reduviidae: Triatomini in 16 homes using an accumulated capture effort of 80 man-hours during dry season and 16 man-hours in the rainy season (Figures 2 and 3). Thirty-five adults, 63 nymphs, and 14 nymph exuviae were collected in various development stages and a wing, all from *T. maculata* (Table 1).

**Table 1:** **Sampling of *Triatoma maculata* (Erichson, 1848) Reduviidae: Triatomini using different trapping methods of Marbacella and El Horno settlements, Riohacha, La Guajira, Colombia 2013**.

Capture method	Positive housing	Marbacella										El Horno									
		Indoor					Outdoor					Indoor					Outdoor				
		*A*			*N*	*R*	*A*			*N*	*R*	*A*			*N*	*R*	*A*			*N*	*R*
		♂	♀	i			♂	♀	i			♂	♀	i			♂	♀	i	
Active search	14	1	0	4	1	2	6	6	0	7	2	2	1	0	0	6	1	0	1	55	5
Occasional capture	2	4	2	0	0	0	4	3	0	0	0	0	0	0	0	0	0	0	0	0	0
Maria biosensors	0	0	0	0	0	0	0	0	0	0	0	0	0	0	0	0	0	0	0	0	0

*A, adults; i, indeterminate sex; N, nymphs; R, exuviae of nymphs and adult triatomines bugs vestiges*.

**Figure 4:**
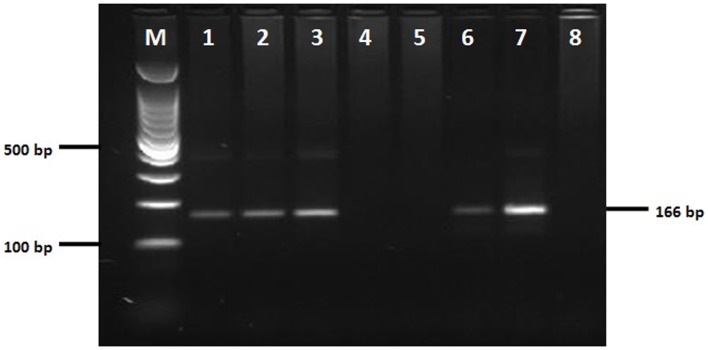
**Detection of DNA of *Trypanosoma cruzi* in insects *Triatoma maculata* by amplification of *T. cruzi* nuclear repetitive region-specific**. Lanes 1–8 are amplification products of tandem repeat satellite region from *T. cruzi* (166 bp). Lanes 1–3: positive samples of *Triatoma maculata* (166 bp). Lanes 4–5: negative samples of *Triatoma maculata*. Lanes 6–7: positive controls of *T. cruzi* (166 bp) Lane 8: negative control. Electrophoresis on a 2% agarose gel visualized by staining with gel red 100-bp weight marker.

Out of 113 collected specimens, 79.6% (*n* = 90) were collected in the peridomicile and 20.3% (*n* = 23) in the intradomicile (Figures 2 and 3), in both cases mainly related to poultry rest and shelter areas and nests. No flagellar parasitic forms to *T. cruzi* were observed in the specimens where inspection by light microscopy (*n* = 16) was performed. However, in 19 of the 32 specimens of *T. maculata* analyzed by molecular biology techniques, a band of 166 bp for the nuclear satellite DNA of *T. cruzi* (Figure 4) was seen. Table 2 shows the vector indices calculated for each indigenous settlement from the data collected.

**Table 2:** **Vector indices of *Triatoma maculata* (Erichson, 1848) Reduviidae: Triatomini in Marbacella and El Horno settlements, Riohacha, La Guajira, Colombia**.

Settlements	Housing inspected	Positive housing	Collection insects						II (%)	PI (%)	HC (%)	VI (%)	VD (%)	DI
			Indoors			Outdoors		
			N	A	R	N	A	R	
Marbacella	51	11	1	11	2	7	19	2	13.7	11.8	9.1	43.5	21.6	0.8
El Horno	17	5	0	3	6	55	2	5	17.6	11.8	20	36	33.3	4.2

*A, adults; N, nymphs; R, exuviae of nymphs and adult triatomines bugs vestiges; II, intradomiciliary infestation indice; PI, peridomiciliary infestation indice; HC, household colonization indice; VI, vector infection indice; VD, vector dispersion indice; DI, vector density indice*.

## Discussion

*T. maculata* is one of the species of the *Triatoma* genus with the greatest geographical distribution in Colombia, being recorded in 11 departments located in the regions of the Orinoco plains and the Caribbean plains (4, 7). The department of La Guajira registers *T. maculata* in the municipalities of Maicao, Barrancas, El Molino, Fonseca, Hato Nuevo, Maicao, San Juan, Urbilla, and Villanueva (7, 8, 16). The results of this study extend and update the geographic distribution of the species in the department, reporting it for the first time in the municipality of Riohacha.

*T. maculata* was the only species recorded in all samples taken in the indigenous settlements of Marbacella and El Horno, a finding that is consistent with that reported in other studies where the predominance of this species is observed in regions with temperatures ranging between 22 and 25°C, rainfall between 1,500 and 2,000 mm annually and vegetation consisting mostly of scrub and thorn (9, 10, 27, 28).

Overall, *T. maculata* showed greater abundance in peridomestic spaces associated with henhouses, pets rest areas, and shelters, with infestation indices identical for both indigenous settlements. This biological preference continues in different geographical locations in Latin America (6, 10, 16, 27, 29–31), which is explained due to the preference for ornithophilic-type eating habits that could be conditioning their biological behavior (32). However, the population density (0.8 for Marbacella and 4.2 for El Horno) and dispersion (21.6 and 33.3, respectively) of the species in this area may be associated with processes of high intraspecific competition due to the search for blood supply and the degree of anthropic disturbance (33), revealing a significant population dynamics resulting in the mobility of the species into different environments.

In Colombia, *T. maculata* was found naturally infected by *T. cruzi* with high infection indices between 50 and 72%; intradomiciliar infestation indices between 13 and 20%, and 38.3% colonization in the municipalities of Talaigua Nuevo and Mompox, department of Bolívar (9, 10). These studies demonstrate the ability of *T. maculata* to infest and colonize stable artificial ecotopes as human habitat and potential efficiency as a vector of Chagas disease.

In this study, exuviae, nymphs, and adults of *T. maculata* were found within households of both indigenous settlements, with colonization indices of 9.1% for Marbacella and 20% for El Horno, which could be suggesting infestation and colonization processes inside the housing. In addition, indices of natural infection with *T. cruzi* between 36 and 43% are reported, which is an important risk factor for the inhabitants of the Wayúu ethnic group in both communities. In contrast to these results, in San Miguel and Xaguas parishes in the Lara State, Venezuela, albeit with similar indices of infestation and colonization, low indices of *T. cruzi* infection were recorded, so that the epidemiological significance of *T. maculata* depends on the environmental, ecological, and socio-cultural factors characteristic of each geographical area (27, 28, 34).

In domiciled triatomines, a relationship is established between housing characteristics and the type of construction material, distribution of peridomestic annexes and finishes of ceilings, walls and floors, as well as socio-cultural practices of its inhabitants with respect to the population distribution and development of triatomine species in homes (35–37). This is of particular importance in both indigenous settlements because due to the physical characteristics of housing (mainly bahareque walls and sand floor) and the use of intradomiciliary spaces as places of poultry refuge and nesting, intrusion, and colonization processes may be assisted by passive transport factors and optimal conditions for the biological development of the species (36, 37).

Ignorance of the local health authorities and communities about the presence and levels of infestation, colonization, and natural *T. cruzi* infection with *T. maculata* and the risk it poses to the people, particularly children, makes it important to strengthen entomological surveillance in the area, together with participation strategies and community action to establish early warning systems for the recognition of the different stages of the insect and the early diagnosis of domiciliation processes of *T. maculata*.

With an aim to the interruption of intradomiciliar transmission of *T. cruzi* with triatomine, Colombia has defined control schemes based on the application of synthetic chemical insecticides in areas of infestation (4). It is important to understand the ecological, biological, and socio-cultural context where vector transmission is occurring and involve affected communities so that strategies for prevention, monitoring, and effective and sustainable control are jointly constructed. The ecobiosocial approach through transdisciplinary research, systems thinking, community participation, and environmental sustainability proves useful for the design, implementation, and evaluation of control strategies appropriate to the ecological, social, and cultural context (38–40). It is suggested to extend these studies in other indigenous settlements, as well as in the urban area of Riohacha because it is possible to find housing conditions and natural ecotopes with appropriate habitats described for this species, which, together with the significant human density in this capital, constitutes a significant risk for Chagas disease. Although in the present study the active search by technical personnel and the community was the most effective method to found exuviae, nymphs, and adults of triatomine, it is important to include in entomological surveillance other strategies based on biosensors (24, 41) and fumigant canisters (41–43) to improve the sensibility of results and its impact in public health policies. In particular, the fumigant canister could be used both to get an idea about infestation indices and to control the insects simultaneously. Nevertheless, it is necessary to inform the community about the risks, benefits, and consequences of the use of insecticide in the fumigant canister.

In conclusion, this is the first report of *T. maculata* naturally infected with *T. cruzi* in the municipality of Riohacha and its geographic distribution in the department of La Guajira has expanded. *T. maculata* was found mainly associated with peridomestic spaces (chicken coops and pens), although indices of intradomiciliar infestation, colonization, and natural infection by *T. cruzi* were found, posing a risk to the Wayúu community of El Horno and Marbacella indigenous settlements. The ethnic and cultural importance of the Wayúu community and the burden that the disease involves in terms of disability and reduced life expectancy (4), makes it necessary for the results to be contextualized as a serious public health problem.

## Authors Contribution

Edith Natalia Gómez-Melendro: development of field and laboratory component. Participation in the preparation of the scientific paper. Diana Carolina Hernández-Castro: development laboratory component and participation in the development of the scientific paper. Catalina Gonzalez-Uribe: principal investigator and participation in the development of the scientific paper. Helena Brochero: principal investigator, coordination of the entomological component and participation in the development of the scientific paper.

## Conflict of Interest Statement

The authors declare that the research was conducted in the absence of any commercial or financial relationships that could be construed as a potential conflict of interest.
